# Vagal afferent fibers contribute to the anti-inflammatory reactions by vagus nerve stimulation in concanavalin A model of hepatitis in rats

**DOI:** 10.1186/s10020-020-00247-2

**Published:** 2020-12-03

**Authors:** Byung Gon Jo, Seung-Hyung Kim, Uk Namgung

**Affiliations:** Department of Oriental Medicine, Institute of Bioscience and Integrative Medicine, Daejeon University, Daehak-ro 62, Daejeon, 34520 South Korea

**Keywords:** Vagus nerve stimulation, Inflammatory cytokines, α7 nicotinic acetylcholine receptor, STAT-3, Capsaicin, Cholinergic anti-inflammation, Liver

## Abstract

**Background:**

Increasing number of studies provide evidence that the vagus nerve stimulation (VNS) dampens inflammation in peripheral visceral organs. However, the effects of afferent fibers of the vagus nerve (AFVN) on anti-inflammation have not been clearly defined. Here, we investigate whether AFVN are involved in VNS-mediated regulation of hepatic production of proinflammatory cytokines.

**Methods:**

An animal model of hepatitis was generated by intraperitoneal (i.p.) injection of concanavalin A (ConA) into rats, and electrical stimulation was given to the hepatic branch of the vagus nerve. AFVN activity was regulated by administration of capsaicin (CAP) or AP-5/CNQX and the vagotomy at the hepatic branch of the vagus nerve (hVNX). mRNA and protein expression in target tissues was analyzed by RT-PCR, real-time PCR, western blotting and immunofluorescence staining. Hepatic immune cells were analyzed by flow cytometry.

**Results:**

TNF-α, IL-1β, and IL-6 mRNAs and proteins that were induced by ConA in the liver macrophages were significantly reduced by the electrical stimulation of the hepatic branch of the vagus nerve (hVNS). Alanine transaminase (ALT) and aspartate transaminase (AST) levels in serum and the number of hepatic CD4^+^ and CD8^+^ T cells were increased by ConA injection and downregulated by hVNS. CAP treatment deteriorated transient receptor potential vanilloid 1 (TRPV1)-positive neurons and increased caspase-3 signals in nodose ganglion (NG) neurons. Concomitantly, CAP suppressed choline acetyltransferase (ChAT) expression that was induced by hVNS in DMV neurons of ConA-injected animals. Furthermore, hVNS-mediated downregulation of TNF-α, IL-1β, and IL-6 expression was hampered by CAP treatment and similarly regulated by hVNX and AP-5/CNQX inhibition of vagal feedback loop pathway in the brainstem. hVNS elevated the levels of α7 nicotinic acetylcholine receptors (α7 nAChR) and phospho-STAT3 (Tyr705; pY-STAT3) in the liver, and inhibition of AFVN activity by CAP, AP-5/CNQX and hVNX or the pharmacological blockade of hepatic α7 nAChR decreased STAT3 phosphorylation.

**Conclusions:**

Our data indicate that the activity of AFVN contributes to hepatic anti-inflammatory responses mediated by hVNS in ConA model of hepatitis in rats.

## Introduction

Vagus nerve is the major parasympathetic nerve connecting the brain to the cardiopulmonary and most of the visceral organs. In addition to its physiological regulatory function, accumulating evidence shows that the vagus nerve activity is involved in mediating neuroimmune interaction (Reardon et al. 2018). The vagus nerve transmits neural activity originated from the central nervous system into the innate and adaptive immune cells in the visceral organs and regulates their pathophysiological function (Tracey 2007; Bonaz et al. 2019). Studies using experimental animals have revealed that the VNS attenuates inflammation in peripheral organs such as spleen and gastrointestinal tracts, and the principles of cholinergic anti-inflammatory pathway in macrophages are widely taken as a mechanistic basis explaining anti-inflammation. For instance, VNS induces JAK2-STAT3 pathway associated with the activation of α7 nicotinic acetylcholine receptor (α7 nAChR) and inhibits the production of pro-inflammatory cytokines such as TNF-α (Wang et al. 2003; de Jonge et al. 2005).

ConA is a lectin that binds to sugar group of glycoproteins on T lymphocytes. In the liver, ConA-bound T cells interact with antigen-presenting cells such as Kupffer cells via MHC-II complex, and consequently, both T cells and Kupffer cells produce inflammatory cytokines and lead to inflammation or cell death in the liver tissue (Tiegs 1997; Krenkel and Tacke 2017). Previous studies have reported that vagus nerve activity is involved in regulating ischemia/reperfusion injury, hepatocyte apoptosis, portal hypertension, inflammatory responses of Kupffer cells, phagocytic activity of macrophages, and oxidative damages in the liver tissues in animal models of liver disease (Zhang et al. 2019a; Özdemir-Kumral et al. 2017; Bockx et al. 2012; Hiramoto et al. 2008; Li et al. 2014; Nishio et al. 2017; Fonseca et al. 2019; Metz and Pavlov 2018). It was further shown that T lymphocytes play a role in modulating the inflammatory responses that are controlled by sympathetic and parasympathetic nerve activities (Wong et al. 2011; Rosas-Ballina et al. 2011), raising the possibility that T lymphocytes activated in ConA-induced hepatitis may be involved in regulating inflammatory responses by VNS.

Given the abundance of afferent fibers accounting 70–80% of the vagus nerve (Prechtl and Powley 1990), VNS applied to cervically exposed nerve may increase the activity of afferent fibers and affect anti-inflammatory responses via contralateral efferent vagus nerve (Inoue et al. 2016). According to cholinergic anti-inflammatory reflex theory, pro-inflammatory cytokines produced from peripheral organs stimulate afferent sensory nerve and consequently augmenting vagal efferent outputs may downregulate inflammatory response (Tracey 2002). However, the contribution of AFVN to VNS-induced anti-inflammation has not been clearly demonstrated in peripheral organs with vagal innervation. To investigate the effects of AFVN on the regulation of inflammatory responses, here we selectively eliminated AFVN by treating with CAP in concanavalin A (ConA)-model of hepatitis in rats. Our study presents new evidence that hVNS-induced activation of AFVN contributes to attenuating the hepatic production of pro-inflammatory cytokines.

## Materials and methods

### Experimental animals and ethical approval

Sprague–Dawley rats (male, 7–8 weeks old, 200–250 g) were purchased (Samtako Inc. Seoul, Korea, RRID:RGD:737,903) and maintained in ventilated animal room with regulated temperature (22–23 °C), 60% humidity under a standard 12 h light and 12 h dark cycle (light on from 7:00 a.m. to 7:00 p.m.). Animals were freely accessed to food pellets (Samyang Co., Seoul, Korea) and water. Total number of animals used for individual experiments are listed in Table 1. No experimental animals died during the experiments and any animals were not excluded in this study from analyses. Animal care and all experimental procedures were in accordance with the NIH Guide for the Care and Use of Laboratory Animals and also approved by the Committee on Use of Live Animals for Teaching and Research at Daejeon University (approval number: DJUARB2019-029, Daejeon, Korea). The authors complied with the ethical principles as outlined in Grundy (2015).

### Drug administration

Rats were anesthetized by inhalation of isoflurane (2%; Hana Pharm Co., Ltd., Seoul, Korea) during CAP or vehicle injection. In order to minimize pain caused by CAP, 6 ml of atropine sulfate (0.5 mg/ml, Jeil Pharmaceutical Co., Daegu, Korea) was injected i.p., 20–30 min prior to CAP injection. CAP (Cayman Chemical Co., Ann Arbor, MI, USA) was dissolved in 10% ethanol, 10% Tween-80 and 0.9% NaCl and was injected i.p. with 40 mg/kg and supplemented with 80 mg/kg twice at 6 h and 24 h after the initial injection as a high-dose regimen (CAP-H) or injected initial 20 mg and supplemented 40 mg/kg twice at 6 h and 24 h later as a low-dose regimen (CAP-L) (Czaja et al. 2008). Volumes of CAP injected to individual animals were evenly adjusted as 1 ml per kg body weight. Equivalent volume of vehicle solution (VEH, 10% ethanol, 10% Tween-80 and 0.9% NaCl) was injected into the control animals. Three days later, animals were subjected to experiments of ConA and hVNS. Efficacy of CAP administration was examined by eye wiping test. CAP was injected into rats with high-dose regimen as described above, and eye wiping behavior was measured 3 days after the initial injection by counting the frequency of eye wiping for 5 min period. Concanavalin A (ConA; 7.5 mg/ml in saline, Sigma-Aldrich, St. Louis, MO, USA) was intravenously injected into the tail with a dose of 15 mg/kg.

In order to inhibit α7 nAChR in the liver, rats were anesthetized with ketamine and (80 mg/kg; Yuhan, Seoul, Korea, Cat #8806421050707) and xylazine (5 mg/kg; Bayer, Leverkusen, Germany, Cat. #KR02315). A combined injection induced a stable anesthetic state for about 1 h, which is an optimal time period to perform the surgery experiment without causing animals’ pain and suffering. The use of ketamine, an antipsychotic drug, has been approved by the Korea Ministry of Food and Drug Administration (Cheongju, Korea, Approval number: DJURFDA-130). Methyllycaconitine citrate salt (MLA; 5 mg/kg in saline, i.p. M168, Sigma-Aldrich), a selective α7 nAChR antagonist was ip injected 20 min prior to hVNS (Tyagi et al. 2010). A mixture (0.5 μl) of MMDA receptor blocker DL-2-amino-5-phosphonopentanoic acid (AP-5, 20 μg/μl, A5282, Sigma-Aldrich) and AMPA receptor blocker 6-cyano-7-nitroquinoxaline2,3-dione (CNQX, 1 μg/μl, C239, Sigma-Aldrich) in saline (0.9%) was bilaterally injected into the area of DMV (coordinate AP: − 14 mm; L: ± 0.6 mm; DV: -8.4 mm) (Paxinos and Watson 1998) with a flow rate of 0.16 μl/min by using a micropump (Pump 11 Elite, Harvard Apparatus, Massachusetts, USA) (Ouagazzal and Amalric 1995; Lim et al. 2016). The injection needle remained penetrated for 3 min after drug injection to prevent inverse flow of injected drugs and also to allow injected drugs to diffuse into the surrounding area. Animals were subjected to hVNS experiment 20 min later.

### Vagus nerve stimulation and hepatic vagotomy

Rats were anesthetized with ketamine and xylazine with the same dose above. The abdomen was incised, the middle, left and right lateral lobes of liver were lifted up, and the stomach was retracted to expose the common hepatic branch of the vagus nerve that is branched out from the ventral vagal trunk (Berthoud and Neuhuber 2000). Common hepatic branch of the vagus nerve is composed of the majority of afferent and minor efferent fibers with myelinated and unmyelinated axons (Prechtl and Powley 1990). We exposed and placed the middle portion of the common hepatic branch of the vagus nerve (~ 2 mm proximal to the location that is divided into hepatic branch proper, portal vein, and gastroduodenal branches) into a U- shape of a bipolar electrode of tungsten wire (250 μm diameter, A-M Systems Inc., Sequim, WA, USA). The electrical current (10 mA, 5 Hz, 5 ms of pulse duration, 5 min) was applied by using the isolated pulse stimulator (model 2100, A-M Systems Inc., Sequim, WA, USA). Our previous study showed that the same stimulation protocol for the acute and chronic VNS at the cervical level was effective to induce the activation of neurons in the dorsal raphe nucleus and the hippocampal neurons in rats (Shin et al. 2019). After hVNS, the abdomen was closed by 5–0 nylon suture, and animals were returned to animal room and sacrificed 1–3 days later. Sham treatment for hVNS was done by exposing the hepatic branch of the vagus nerve in anesthetized rats and suturing the skin without applying electrical stimulation. Animals underwent the same recovery procedure as hVNS group animals. The animals were sacrificed with an overdose of ketamine (150 mg/kg, i.p.). Vagotomy of the hepatic vagal branch (hVNX) in rats was conducted as described previously (Izumi et al. 2018). Briefly, abdominal wall was excised and hepatic branch of the vagus nerve was exposed beneath the liver by using the same procedure as described above. Immediately after cutting around the middle portion of the hepatic vagus nerve, the distal portion of the cut nerve was carefully placed into a bipolar hook electrode of tungsten wire (250 μm diameter, A-M Systems Inc., Sequim, WA, USA). The location of electrical stimulation and stimulation intensity were essentially the same as described above. Abdominal wall was sutured and animal was sacrificed 24 h later.

### Retrograde tracing

In order to identify vagal sensory neurons in the nodose ganglion (NG) innervating the liver tissue in rats, we performed a retrograde tracing experiment. Animals were anesthetized with ketamine (80 mg/kg) and xylazine (5 mg/kg), and 1,1′-Dioctadecyl-3,3,3′,3′-tetramethylindocarbocyanine perchlorate (DiI, 3 μl of 0.5% in DMSO, Sigma-Aldrich) was taken by using Hamilton Syringe (#80330, Reno, NV, USA) and slowly applied to the cut end of the hepatic branch of the vagus nerve for 2–3 min. After suturing incised abdomen skin tissue, animals were recovered from narcosis and maintained in animal room for 7 days until the dissection of NG for further analysis. To dissect NG ganglion, we cut the anterior surface of the neck and exposed the carotid artery. The vagus nerve was carefully exposed from the carotid artery by removing muscles and connective tissues along the rostral direction. The NG was isolated just above the location where the hypoglossal nerve crosses the vagus nerve.

### Western blot analysis

Liver tissue was dissected from rats and sonicated in RIPA buffer (150 mM NaCl, 1.0% CA-630, 0.1% SDS, 50 mM Tris, pH 8.0, 0.5% sodium deoxycholate; Thermo Fisher Scientific, Waltham, MA, USA) supplemented with protease inhibitor and phosphatase inhibitor cocktails (Roche Diagnostics, Canton, Switzerland). The lysate was centrifuged at 12,000 rpm, 15 min, and 4 °C and the supernatant was collected. Extracted proteins (20 μg) were separated by SDS–polyacrylamide gel electrophoresis (SDS-PAGE) and transferred to PVDF membrane. PVDF membrane was placed in blocking solution (5% BSA, 1× TBST (0.1% tween 20 in Tris-buffered saline)) and followed by primary and secondary antibody reactions. For the quantitative analysis of ChAT protein in the NTS and DMV area, coronal brain sections (60 μm thickness) were prepared by using a cryostat (CM1850, Leica, Wetzlar, Germany). Sections were placed on the slides and the area covering NTS and DMV were scrapped off under the stereoscopic microscope by using 3 ml syringe. Preparation of cell lysates and remaining steps of western blotting experiment were essentially the same as described previously (Chang et al., 2012). Immunoblotting was performed with primary antibodies against TNF-α (ab9739, Rabbit-polyclonal, 1:2,000, Abcam, Cambridge, UK, RRID:AB_308774), IL-1β (ab9722, Rabbit-polyclonal, 1:2,000; Abcam, RRID:AB_308765), IL-6 (ab9324, Mouse-monoclonal, 1:2000; Abcam, RRID:AB_307175), ChAT (ab181023, Rabbit-monoclonal, 1:2000, Abcam, RRID:AB_2687983), pY-STAT3 (#9145, Rabbit-polyclonal, 1:1000; Cell Signaling Technology, Danvers, MA, USA, RRID:AB_2491009), α7 nAChR (ANC-007, Rabbit-polyclonal, 1:200, Alomone Labs, Jerusalem, Israel), and β-actin (A1978, Mouse-monoclonal, 1:50,000; Sigma-Aldrich, RRID:AB_476692). Secondary antibodies were anti-rabbit IgG HRP (#7074, 1:5000; CST, RRID:AB_2099233) and anti-mouse IgG HRP (#7076, 1:5000; CST, RRID:AB_330924) antibodies. Intensity of protein bands in the X-ray film was determined by densitometric measurement using the i-Solution software (http://www.imt-digital.com/English2.0/html/home.php, version 21.1, Image & Microscope Technology, Daejeon, Korea).

### Real-time PCR and RT-PCR

Total RNA was extracted from the liver and brainstem tissues by using trizol reagent (Thermo Fisher Scientific). cDNA was synthesized by incubating isolated RNA in the reaction containing 50 mM Tris–HCl, 3 mM MgCl_2_, 75 mM KCl, 10 mM DTT), 104 μM dNTP mixture, RNasin (30 U), random primers (16 μM, Promega, Madison, WI, USA), and MMLV reverse transcriptase (200 U, Promega) for 2 h at 37 °C. RT-PCR was performed by using Green Master Mix (Promega) as described previously (Chang et al. 2018). The primer sequences for RT-PCR are as follows; the forward primer (5′-TTCTTTGTCTTGGATGTTGTCAT-3′) and reverse primer (5′-AACATTTCAACCTCAACCTTCTGG-3′) for ChAT mRNA, the forward primer (5′-TGTAGGCCTGCTGGATCAAC-3′) and reverse primer (5′-GCAGGATATCAGCTCGGTGT-3′) for AChE mRNA, and the forward primer (5′-CACACTGTGCCCATCTATGA-3′) and the reverse primer (5′-GCAGGATATCAGCTCGGTGT) for actin mRNA. Amplified DNA was analyzed by 1% agarose gel electrophoresis. Real-time PCR was performed in a 20 μl reaction volume containing synthetic cDNA (3 μg), 1× Power SYBR Green PCR Master mix (Life technologies, Carlsbad, CA, USA), and 0.15 μM forward and reverse TNF-α primers. PCR for rat GAPDH gene was carried out by incubating synthesized cDNA (3 μg), 1× TaqMan Gene Expression Master Mix (AmpliTaq Gold DNA Polymerase, Thermo Fisher Scientific), 1× rat GAPDH primer (Rat GAPD, Applied Biosystems, Foster City, CA, USA), and endogenous control (VIC1/MGB probe primer) in a 20 μl of reaction volume. Real-time PCR reactions were performed using 96 well plate (MicroAmp Optical 96-well reaction plate, Applied Biosystems). The plate was covered with a film (MicroAmp Optical Adhesive Film, Applied Biosystems) and centrifuged briefly to spin down the sample (GS-6R Centrifuge, Beckman Coulter Life Sciences, Brea, CA, USA). Real-time PCR was carried out using an 7500 Real Time PCR System (Applied Biosystems) by activating Taq polymerase at 50 °C for 2 min and at 95 °C for 10 min, followed by 40 cycles with 15 s at 95 °C for denaturation and 1 min at 60 °C for annealing. The RNA levels in each group were represented as fold changes in levels of target mRNAs to GADPH reference mRNA. The primer sequences for real-time PCR were as follows; the forward primer (5′-ACAAGGCTGCCCCGACTAT-3′) and the reverse primer (5′-CTCCTGGTATGAAGTGGCAAATC-3′) for TNF-α mRNA, the forward primer (5′-GGGCGGTTCAAGGCATAACAG-3′) and the reverse primer (5′-CTCCACGGGCAAGACATAGG-3′) for IL-1β mRNA, the forward primer (5′-CTGGTCTTCTGGAGTTCCGT-3′) and the reverse primer (5′-TGGTCCTTAGCCACTCCTTCT-3′) for IL-6 mRNA, and the forward primer (5′-GACCCAGAAGCTTCCAAGCCA-3′) and the reverse primer (5′-TGGGCATTGTAGTGACTCTCG-3′) for ChAT mRNA. The relative quantification (RQ) value of TNF-α, IL-1β, IL-6 and ChAT mRNA expression on GAPDH mRNA was calculated by the threshold cycle (Ct) data.

### Immunofluorescence staining

Rats were anesthetized with overdose of ketamine and xylazine and perfused by using 4% paraformaldehyde in 1× PBS. Tissues such as brainstem, NG, and liver were dissected and immersed overnight in 20% sucrose in PBS solution. After rapid freezing with − 80 °C of dimethylbutane (Sigma-Aldrich), tissues were cut using a cryostat (Leica, Wetzlar, Germany) and thaw-mounted on the slide (16 μm thickness). Immunofluorescence staining was performed as described previously (Chang et al. 2012). Briefly, sections were fixed, permeabilized, treated with blocking solution (2.5% BSA and 2.5% horse serum, 0.1% Triton X-100 in 1× PBS), and incubated with primary antibodies for 24 h at 4 °C, washed three times with 1× PBST and incubated with secondary antibodies at room temperature for 2 h in a dark room. The primary antibodies used were anti-TNF-α (ab9739, Rabbit-polyclonal, 1:400; Abcam), anti-IL-1β (ab9722, Rabbit-polyclonal, 1:400; Abcam), anti-IL-6 (ab9324, Rabbit-polyclonal, 1:400; Abcam), anti-P2X2 (PA1-24624, Rabbit-polyclonal, 1:400; Thermo Fisher Scientific, Waltham, MA, USA, RRID:AB_2157912), anti-c-Fos (sc-166940, Mouse-monoclonal, 1:400; Santa Cruz Biotech, Dallas, Texas, USA, RRID:AB_10609634), anti-cleaved Caspase-3 (#9661, Rabbit-polyclonal, 1:400; CST, RRID:AB_2341188), anti-CD11b (554980, Mouse-monoclonal, 1:200; BD Biosciences, Franklin Lakes, NJ, USA, RRID:AB_2129492), anti-pY-STAT3 (#9145, Rabbit-polyclonal, 1:400; CST), anti-NF-200 (N0142, Mouse-monoclonal, 1:400; Sigma-Aldrich, RRID:AB_477257), anti-ChAT (ab181023, Rabbit-monoclonal, 1:400, Abcam), anti-VR1 (M-1714-100, Mouse-monoclonal, 1:400, Abcam, RRID:AB_2492520), anti-Albumin (NBP1-32458, Rabbit-polyclonal, 1:200, Novus Biologicals, Centennial, CO, USA, RRID:AB_10003946), normal mouse IgG (sc-2025, Mouse-monoclonal, 1:400, Santa Cruz Biotechnology, RRID:AB_737182) and normal rabbit IgG (#2729, Rabbit-polyclonal, 1:400, CST, RRID:AB_1031062) antibodies. Rhodamine-goat anti-rabbit IgG (R-6394, 1:400; Molecular Probes, Eugene, OR, USA, RRID:AB_2556551) and fluorescein-goat anti-mouse IgG (F-2761, 1:400; Molecular Probes, RRID:AB_2536524) antibodies were used as secondary antibodies. When necessary for nuclear staining, sections were incubated with Hoechst 33258 (2.5 μg/ml, bis-benzimide, Sigma) for 10 min before the final washing with 1× PBST. Signal intensity of immunofluorescence images were measured by using the program Image J (ImageJ, NIH, Bethesda, MA, USA, RRID:SCR_003070). Quantification was represented as either the signal intensity or the number of cells displaying effective pixel values. Fluorescence images were converted into grayscale mode and the pixel density above the threshold which had been set in the program was adapted as being effective for further quantification. Fluorescence intensity in the images was presented as the pixel density relative to that of the control images. Also, individual cells showing the pixel density above the threshold which had been set in the same way as above were counted. The number of labeled cells or the pixel density in the field of image were counted and averaged for 3–5 nonconsecutive sections. Observers were blind to the slides that were used for the analysis by fluorescence microscopy.

### Flow cytometry

FACS analysis in liver tissue was conducted as described previously with some modifications (Saba et al. 2020). Briefly, rats were anesthetized by ketamine and xylazine and the blood was removed by cardiac puncture. The equal amount of liver tissue (0.2 g) was dissected from individual animals. The liver tissue was chopped into small pieces and treated with 3 ml of type IV collagenase (10 μg/ml reaction, Sigma) in 2% FBS and 1 mM EDTA at 37 °C for 30 min. The tissue was homogenized by using gentleMACS dissociator (Miltenyi Biotech, Bergisch Gladbach, Germany) and filtrated through a 70 μm pore size nylon cell strainer (BD Falcon, Bedford, MA, USA). The filtrated cells were centrifuged at 300×*g* for 10 min and the pellets were incubated for 2 min in 3 ml of ACK solution (0.15 M NH_4_Cl, 1 mM KHCO_3_, 0.1 mM EDTA) to lyse erythrocytes. After washing with FACS buffer (2% FBS in 1× PBS; Gibco-RBL, Grand Island, NY, USA) and centrifugation, pellets were resuspended in a fresh FACS buffer and transferred to FACS tube (Corning, New York, NY, USA). The aliquots were taken to measure the number of cells by using haemocytometer (DHC-N01, INCYTO Co., Ltd., Cheonan, Korea). Cells were pelleted by centrifugation at 390×*g* for 5 min and resuspended in 10 μl of blocking buffer (5% BSA in 1× PBS). Cells were incubated with antibodies for 30 min at 4 °C. After washing with FACS buffer and centrifugation for 5 min at 390×*g*, cell pellets were suspended in 300 μl of fix buffer (4% paraformaldehyde in 1× PBS) and stored at 4 °C until the analysis with two-colour flow cytometry on a BD FACSCaliburTM. The data were analyzed using CellQuest software (BD Biosciences, 209 Mountain View, CA, USA). Monoclonal antibodies used in the present study were anti-CD3-fluorescein isothiocyanate (FITC, 1:10, 557354, BD Biosciences, San Diego, CA, USA), anti-CD8-phycoerythrin-PE (PE, 1:10, 559976, BD Biosciences), anti-CD4-PE (1:10, 554836, BD Biosciences), and anti-CD161-FITC (1:10, 205608, BioLegend, San Diego, CA, USA) primary antibodies.

### AST and ALT measurement in serum

Blood was collected from the cardiac puncture in rats, centrifuged at 1157×*g* for 4 °C, and the serum in the upper phase was taken. The levels of AST and ALT in serum were analyzed by a protocol as provided by the manufacturer (ASAN pharmacy, Seoul, Korea). Briefly, a mixture (100 μl) of l-aspartate (22.6 mg/l) and α-ketoglutarate (292 mg/l) or dl-alanine (17.8 mg/ml) and α-ketoglutarate (292 mg/l) was preincubated for 5 min at 37 °C, added serum (20 μl), and incubated 30 min at 37 °C. After adding 100 μl of 2,4-dinitrophenylhydrazinbe (198 mg/l) and incubating for 20 min at 37 °C, we stopped the reaction with 1 ml of NaOH (0.4 N) for 20 min at room temperature. The sample was then used for spectrophotometric measurement at 505 nm and the values of absorbance were converted into Karmen Unit (IU/l).

## Experimental design and statistical analysis

For the studies examining the effects of hVNS on the production of inflammatory cytokines, rats were randomly assigned to untreated control (CTL), ConA + Sham, and ConA + hVNS. Sham or hVNS treatments were performed in ConA-injected animals. Liver and brainstem tissues were collected for RT-PCR, western blotting, flow cytometry, and immunofluorescence experiments. In the other set of experiments, ConA + hVNS animals were randomly assigned into the subgroups of CAP or sham treatment, AP-5 and CNQX injection, and hVNX surgery. Some of CAP-injected rats and untreated control rats were used to examine eye wiping test. The experimenter was blinded to the animal’s groups during experimentation and statistical analysis.

All data were presented as mean ± standard deviation (SD). The mean number data among experimental groups were compared by using Student’s t test (unpaired) or one-way ANOVA and Tukey’s multiple comparison test for multiple comparisons (GraphPad Prism 7.00, GraphPad Software Inc., San Diego, CA, USA). Statistically significant differences were set at *p < 0.05, **p < 0.01, ***p < 0.001.

## Results

### hVNS downregulates hepatic expression of inflammatory cytokine mRNAs and proteins in ConA-injected animals

**Fig. 1:**
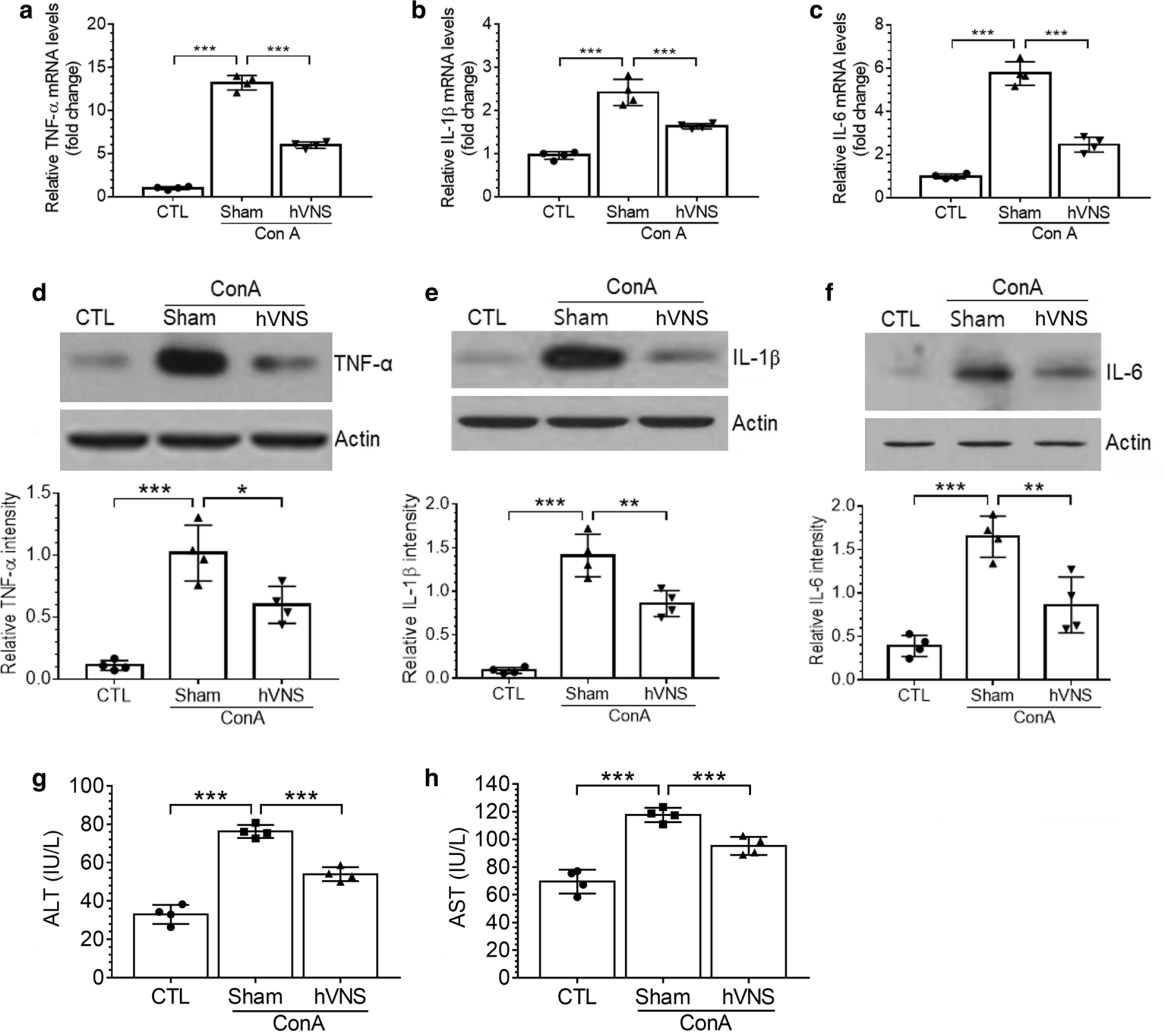
Down-regulation of the production of inflammatory cytokine mRNAs and proteins by hVNS in ConA-injected animals. hVNS was administered 24 h after ConA injection and the liver tissue was dissected for real-time PCR and western blot analyses 24 h later. **a**–**c** Quantification of mRNAs for TNF-α, IL-1β, and IL-6 by real-time PCR. Levels of target mRNA in ConA + Sham and ConA + hVNS groups relative to untreated control (CTL) are plotted. **d**–**f** Western blotting of TNF-α, IL-1β, and IL-6 proteins in the liver tissue. Upper images in **d**–**f** are the representatives from four independent experiments and the plots below present quantitative comparison of band intensity relative to actin. Western blotting for actin in **d**–**f** as sample loading control. **g**, **h** ALT and AST levels in serum of CTL, ConA + Sham, and ConA + hVNS groups of animals. In **a**–**h**, one-way ANOVA with Tukey post hoc is shown. *p < 0.05, **p < 0.01, ***p < 0.001

In the present study, we applied VNS to the hepatic branch of the vagus nerve in order to study the effect of the electrical stimulation on the anti-inflammatory reflex via the feedback interaction between the hepatic afferent and efferent fibers of the vagus nerve, while minimizing physiological interference to other visceral organs and cardiovascular systems. To investigate whether hVNS has the effects on the inflammatory responses in the liver tissue, we delivered hVNS 24 h after ConA injection and analyzed the hepatic expression of TNF-α, IL-1β and IL-6 inflammatory cytokines 24 h later. mRNA levels of TNF-α, IL-1β, and IL-6 were significantly elevated by ConA treatment and down-regulated by hVNS (Fig. 1a–c). Similarly, protein levels of TNF-α, IL-1β, and IL-6 were markedly increased in the liver tissue after ConA treatment but down-regulated by hVNS (Fig. 1d–f). As pathological indicator of hepatitis, we analyzed ALT and AST. As shown in Fig. 1g, h, ALT and AST levels in serum were significantly increased after ConA treatment and decreased by hVNS.

**Fig. 2:**
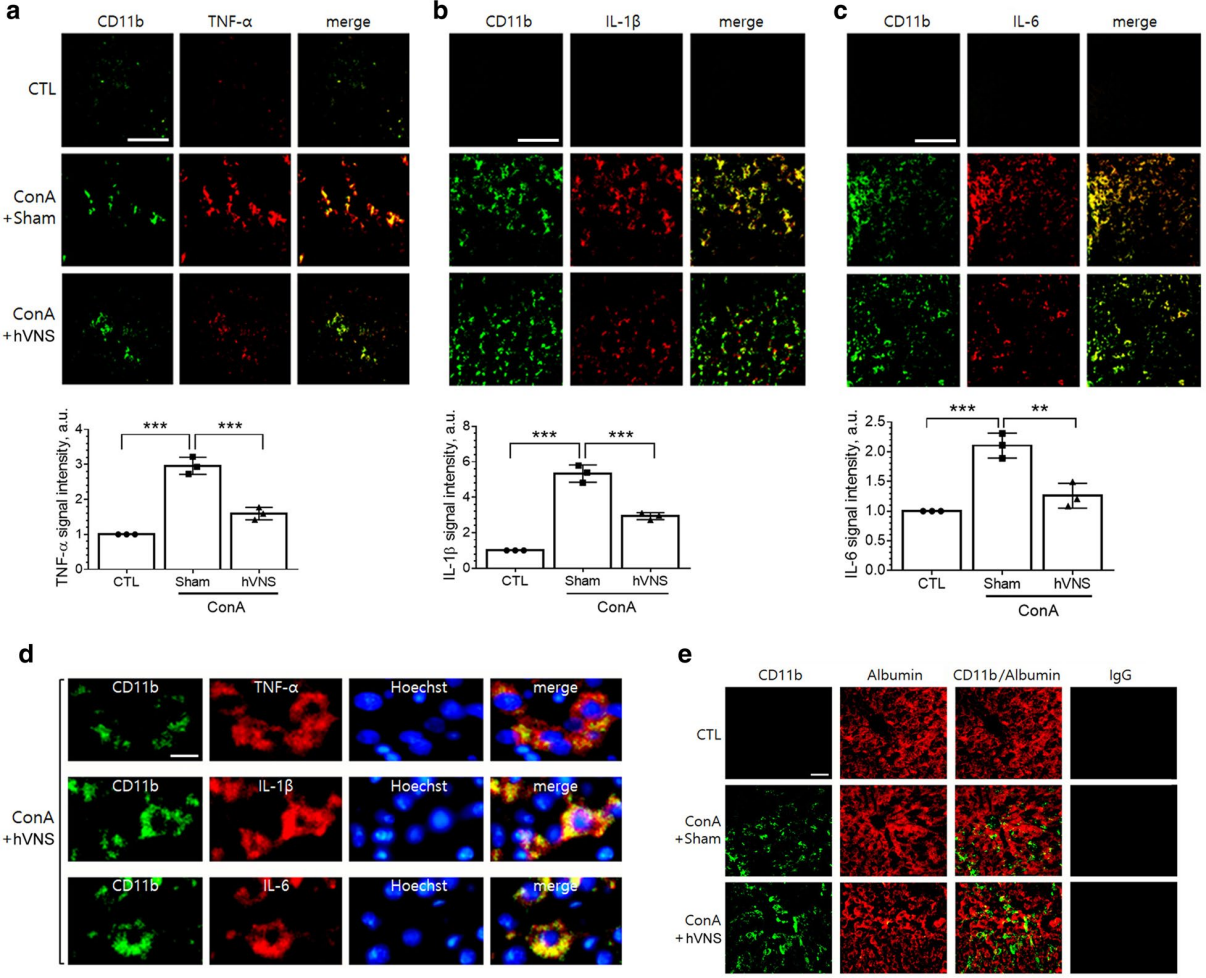
Immunofluorescence localization of TNF-α, IL-1β and IL-6 in the liver tissue of animals treated with ConA or ConA + hVNS. **a**–**c** Representative immunofluorescence images of TNF-α, IL-1β, and IL-6 that were merged with CD11b. Signal intensities of TNF-α, IL-1β, and IL-6 from 4 to 6 images were averaged per each animal and compared among experimental groups (n = 3 independent experiments). One-way ANOVA with Tukey post hoc is shown. *p < 0.05, **p < 0.01, ***p < 0.001. **d** Enlarged views of TNF-α, IL-1β, and IL-6 signals merged with CD11b and Hoechst nuclear staining images in the liver sections from ConA + hVNS animal. **e** Immunofluorescence staining of liver tissue with anti-CD11b and anti-albumin antibodies. Representative immunofluorescence images using normal mouse IgG antibody instead of primary antibodies followed by reaction with fluorescein-goat anti-mouse IgG antibody are shown in the last column. Scale bars in **a**–**c**, **d**, and **e** are 100 μm, 10 μm, and 50 μm respectively

To determine cell types producing inflammatory cytokines, we performed immunofluorescence staining of liver tissue. Signals of TNF-α, IL-1β, and IL-6 were clearly induced by ConA treatment, being mostly colocalized with macrophage marker protein CD11b which was also increased by ConA (Fig. 2a–c upper panel). Then, the signals of TNF-α, IL-1β and IL-6 were reduced by hVNS in ConA-treated animals. Quantitative measurement of protein signals showed significant increases by ConA and decreases by hVNS (Fig. 2a–c lower panel). The protein signals, colocalized with CD11b in ConA-treated animals, were detected in perinuclear area, as identified by merged view with Hoechst nuclear staining (Fig. 2d). We also examined the reaction specificity of CD11b antibody by co-immunolabeling with albumin protein that is expressed only in parenchymal hepatocytes (Gu et al. 2017). CD11b-labeled cells observed from ConA + Sham and ConA + hVNS groups were clearly distinguished from the outnumbered albumin-labeled hepatocytes (Fig. 2e). No signals were detected by normal IgG antibody reactions in all experimental groups, supporting the specificity of immunolabeling reactions.

**Fig. 3:**
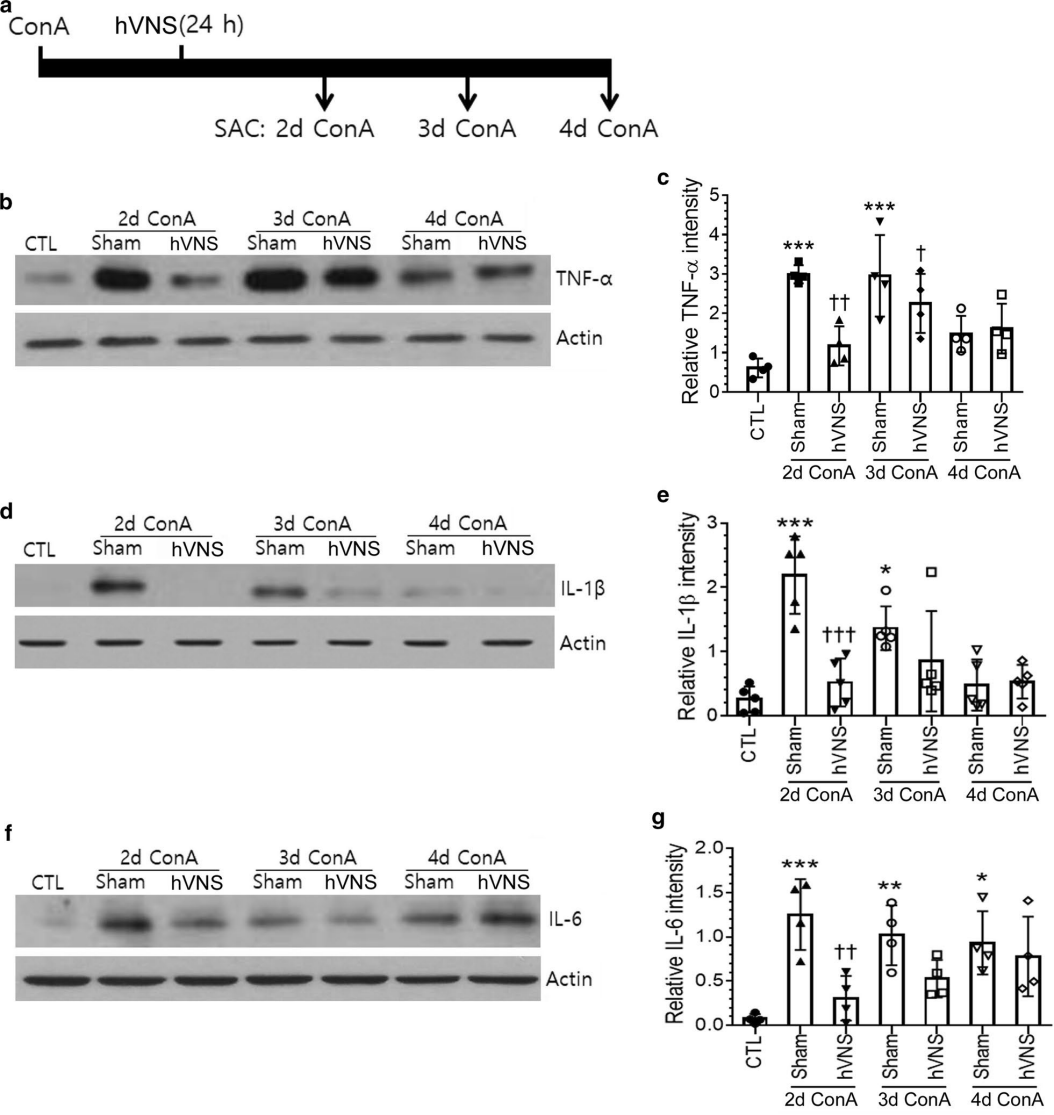
Changes in inflammatory cytokine levels in the liver tissue of ConA-injected animals at different time points after hVNS. **a** A schematic showing the time schedule for the treatments of ConA and hVNS and a sacrifice (SAC) of animals for western blot analysis. Animal groups are named based on the time interval between ConA injection and SAC. **b**, **c** Western blot analysis of TNF-α for the liver lysates from individual group of animals. **d**, **e** Western blot analysis of IL-1β for the liver lysates from individual group of animals. **f**, **g** Western blot analysis of IL-6 for the liver lysates from individual group of animals. Images in **b**–**f** are the representatives from four to five independent experiments, and the quantifications of protein band intensity relative to actin are plotted in **c**, **e**, and **g**. Western blotting for actin as sample loading control. In **c**, **e** and **g**, one-way ANOVA with Tukey post hoc is shown. In **c**, ***p < 0.001 vs. CTL, †p < 0.05 vs. 3d VNS + Sham, ††p < 0.01 vs. 2d VNS + Sham. In **e**, *p < 0.05 vs CTL, ***p < 0.001 vs. CTL, †††p < 0.001 vs. 2d VNS + Sham. In **g**, *p < 0.05 vs. CTL, **p < 0.01 vs. CTL, ***p < 0.001 vs. CTL, ††p < 0.01 vs. 2d VNS + Sham

To investigate the persistency of anti-inflammatory effects of hVNS in ConA-treated liver tissue, we analyzed levels of inflammatory cytokines 2–4 days after ConA injection (; individual groups labeled 2d, 3d, and 4d ConA groups in Fig. 3a). Levels of TNF-α, IL-1β, and IL-6 were maintained or partly reduced in 2d and 3d ConA groups of animals and further decreased in 4d ConA group (Fig. 3b, d, f). Vagal modulation of inflammatory cytokines, in terms of the decreases in cytokine levels in the presence of hVNS, was obvious in 2d ConA groups and became less effective in 3d and 4d ConA groups (Fig. 3c, e, g). In 3d ConA groups, levels of three cytokines were all decreased after hVNS administration though the changes were not statistically significant in IL-1β, and IL-6 (% decreases and p values; 63% and p = 0.58 for IL-1β, 52% and p = 0.35 for IL-6).

### hVNS reduces the recruitment of immune cells in the liver of ConA-injected animals

**Fig. 4:**
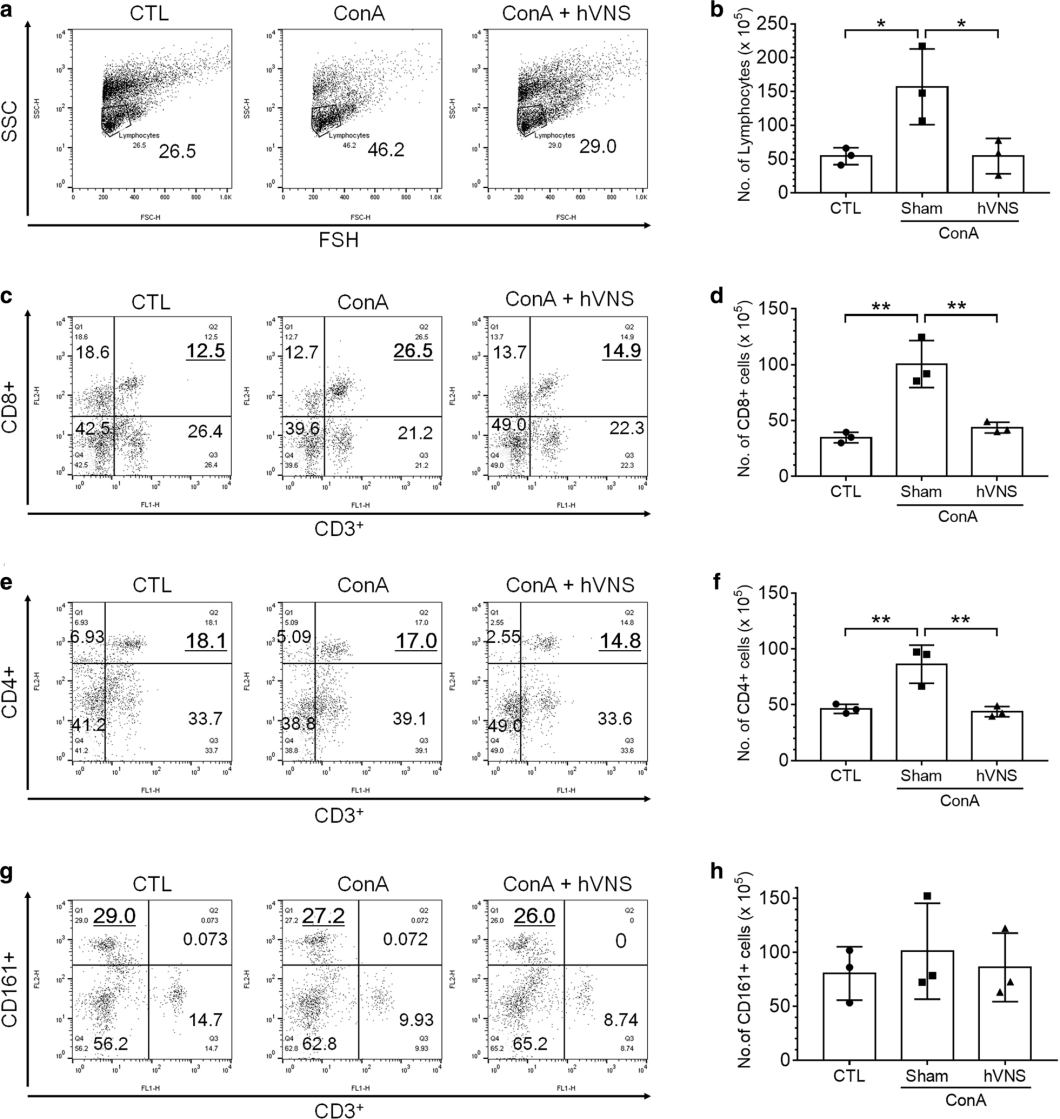
Down-regulation in infiltration of CD8^+^ and CD4^+^ lymphocytes by hVNS in Con A-injected animals. Flow cytometry analysis were gated on FSH vs. SSC for lymphocyte (**a**, **b**), CD3^+^ and CD8^+^ for CD8^+^ T cells (**c**, **d**), CD3^+^ and CD4^+^ for CD4^+^ T cells (**e**, **f**), and CD3^+^ and CD161^+^ for NK cells (**g**, **h**). Quantification of absolute cell numbers was plotted and compared among experimental groups (**b**, **d**, **f**, **h**). The underlined numbers in the quadrants in **c**, **e**, **g** denote the percentage of target cells used for counting. In all plots in **b**, **d**, **f**, **h**), one-way ANOVA with Tukey post hoc is shown. *p < 0.05, **p < 0.01 (n = 3)

To measure the changes in the number of total lymphocytes, CD8^+^ and CD4^+^ T cells, and natural killer (NK) cells, we analyzed intrahepatic cells by flow cytometry. The percentage of lymphocytes was gated on FSH versus SSC, and absolute number of lymphocytes was calculated by multiplying the percentage value and the total cell counts. The percentage and number of lymphocytes were increased by ConA treatment and decreased by VNS (Fig. 4a, b). Similarly, the percentage and number of CD8^+^ T cells (CD3^+^ CD8^+^) were increased by ConA and downregulated by hVNS (Fig. 4c, d). The percentage of CD4^+^ T cells (CD4^+^ CD3^+^) was similar between control and ConA groups, but the absolute number of CD4 + T cells was increased by ConA and downregulated by hVNS (Fig. 4e, f). The percentage and number of CD161^+^ NK cells did not vary among three groups (Fig. 4g, h).

### hVNS increases the expression of ChAT mRNA and protein in DMV neurons

**Fig. 5:**
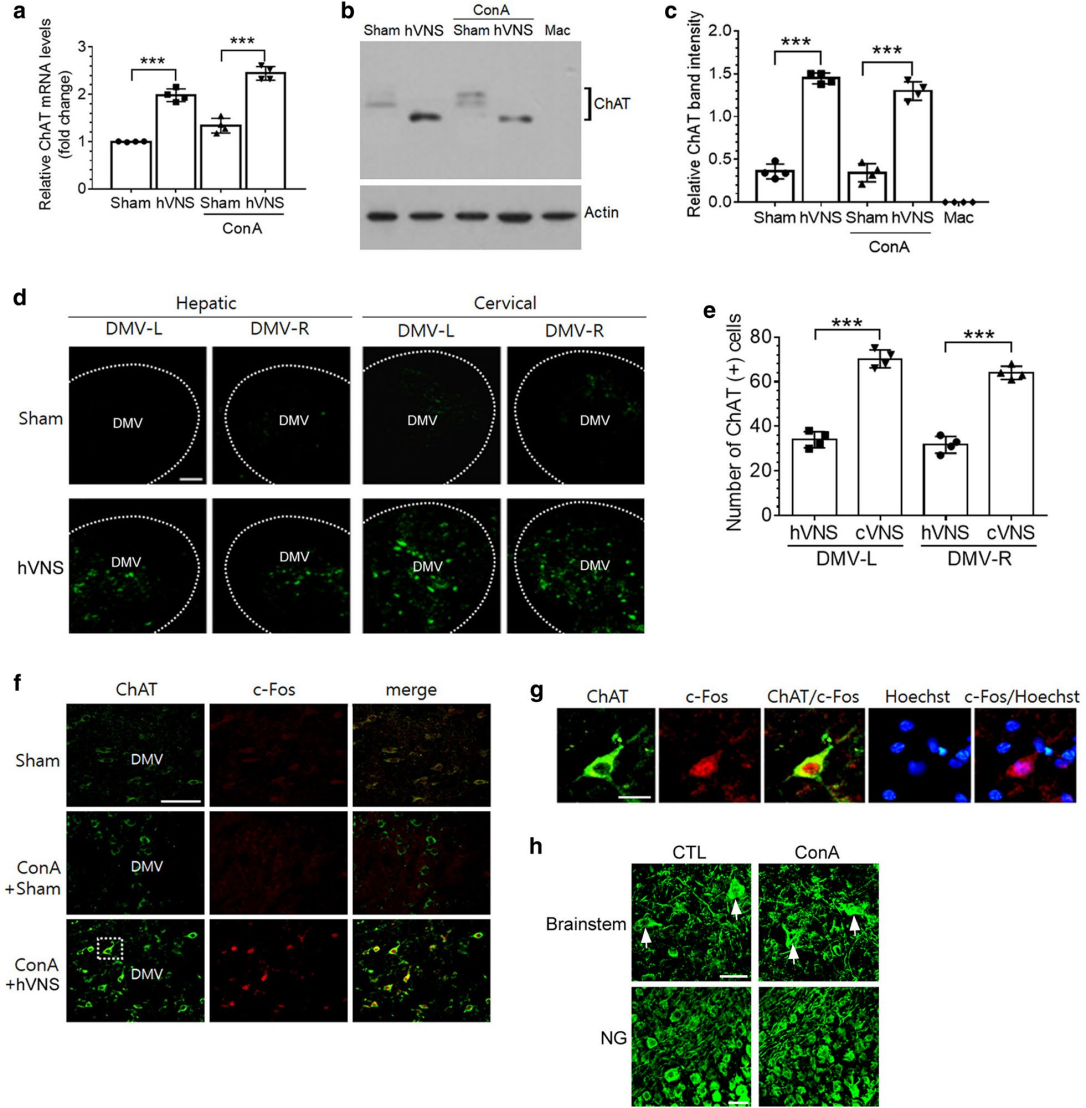
Induction of ChAT expression by hVNS in DMV brain tissues. **a** Quantification of ChAT mRNA by real-time PCR. **b**, **c** Western blot analysis of ChAT protein. Images in **b** are the representative from four independent experiments. Quantification of ChAT protein band intensity relative to actin is plotted in **c**. **d**–**g** Immunofluorescence staining of ChAT/c-Fos proteins in the brain tissues. **d** Representative images showing ChAT signals in the left (L) and right (R) sides of DMV areas in groups of animals given VNS at the hepatic branch or at the cervical location along with corresponding Sham treatments. **e** Quantification of the number of ChAT-positive cells in the DMV after hepatic VNS (hVNS) and cervical VNS (cVNS). **f** Immunofluorescence images of ChAT/c-Fos in the brainstem sections. Both ChAT and c-Fos signals were clearly detected from neurons in the left side of DMV of ConA + hVNS group of animals. **g** Enlarged images of the dotted rectangular area of ConA + hVNS animal in **f** showing the colocalization of ChAT and c-Fos signals. Note intense c-Fos signal in the nucleus identified by Hoechst staining (blue). **h** Immunofluorescence staining of neurons in the DVC area in the brainstem and the nodose ganglion (NG) with anti-NF-200 antibody. **a**, **c**, **e**, one-way ANOVA with Tukey post hoc is shown. ***p < 0.001 (n = 4). Scale bars in **d **and **f**, **g**, and **h** are 100 μm, 20 μm, and 50 μm, respectively

ChAT catalyzes the synthesis of acetylcholine in the presynaptic terminal of cholinergic neurons and its expression is induced in glutamate-applied cholinergic neurons and splenic T cells after VNS (Rosas-Ballina et al. 2011; Zhou et al. 2008). Here, we examined the expression of ChAT mRNA and protein in the DMV neurons. hVNS induced significant increases in ChAT mRNA level in both non-ConA and ConA-treated groups (Fig. 5a). However, ConA treatments in Sham control and hVNS groups did not show significant increases in ChAT mRNA compared to their corresponding non-ConA groups. Western blot analysis showed that ChAT proteins were similarly increased by VNS (Fig. 5b) and quantitative comparison revealed significant increases in band intensity by hVNS in both non-ConA and ConA treated groups (Fig. 5c). ChAT proteins were detected as multiple bands between 67–72 kDa with higher molecular weights in Sham and ConA + Sham groups than those in corresponding hVNS-administered groups. We detected no protein band from the cell lysates of the macrophages that have shown no expression of ChAT in the previous report (Fujii et al. 2017), supporting the reaction specificity of ChAT antibody. Immunofluorescence analysis showed that in animals given hVNS (; hepatic VNS), the number of ChAT-positive cells (green signals in Fig. 5d, f, g) were slightly higher in the ipsilateral side (left DMV; DMV-L) than the contralateral side (right DMV; DMV-R), yet failing to show statistical difference. (11.7% difference with p = 0.5, One-way ANOVA; Fig. 5e). ChAT expression in the DMV induced by hVNS was lower in the number and more restricted in its distribution, compared with the pattern of ChAT expression after VNS at the cervical location (labeled cVNS in Fig. 5d, e), indicating the suborganization of ChAT-expressing DMV neurons that respond to hVNS. Moreover, ChAT protein, showing intense signals in ConA + hVNS group compared to Sham control and ConA groups, was mostly colocalized with c-Fos protein in the DMV neurons (Fig. 5f). Enlarged image revealed that c-Fos proteins displaying clear signal in the nuclear zone were colocalized with cytoplasmic ChAT-labeled neurons in the DMV (Fig. 5g). We also examined whether ConA injection into the rat affected neuronal integrity in the nervous system as a consequence of possible hepatotoxic activity caused by ConA. Immunofluorescence analysis showed that the organization of neurons in the brainstem and in the NG showed the normal pattern of neuronal cell body and processes similarly in both untreated control and ConA-treated animals (Fig. 5h; distinct neuronal cell bodies in the brainstem marked by arrows).

### CAP degenerates NG sensory neurons

**Fig. 6:**
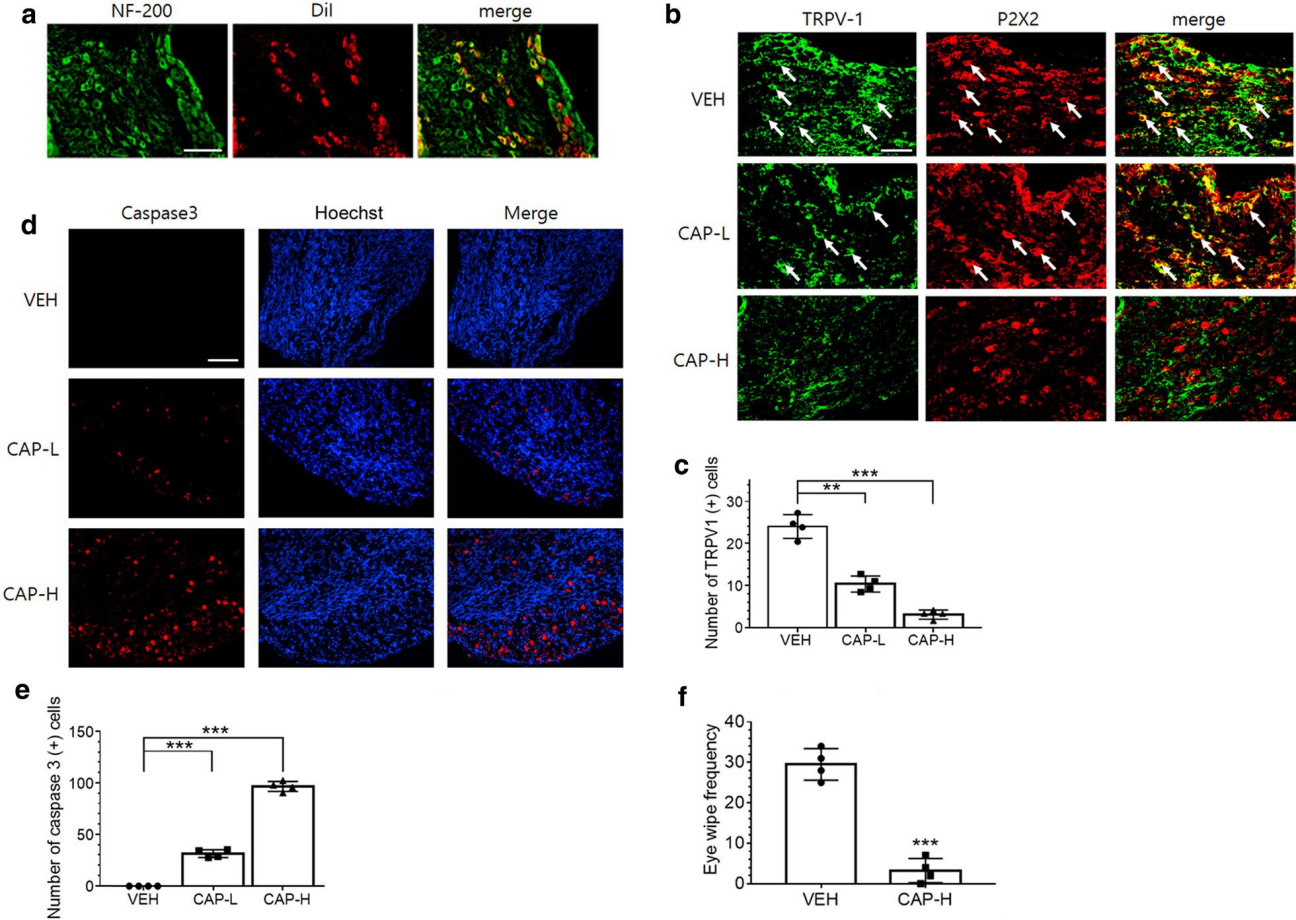
Immunofluorescence identification of degenerating NG neurons in CAP-treated animals. **a** Colocalization of DiI-labeled neurons with NF-200-immunostained neurons in the NG. Seven days after DiI injection into the hepatic vagal branch in rats, NG was dissected and subjected to immunofluorescence staining for NF-200. **b**–**e** Immunofluorescence staining of TRPV1 and P2X2 proteins in the NG. Three days after CAP injection with a total of 100 mg/kg (CAP-L) or 200 mg/kg (CAP-H), NG sections were prepared for immunofluorescence staining for TRPV1 and P2X2 receptor proteins (**b**) or for cleaved form of caspase 3 with Hoechst 33258 nuclear staining (**d**). The number of neurons labeled with TRPV1 and caspase 3 proteins were quantified and plotted in **c** and **e**, respectively. **p < 0.01, ***p < 0.001 (One way ANOVA with Tukey post hoc, n = 4). Scale bars in **a**, **b**, and **d** are 100 μm. **f** Eye wiping test in rats. Eye wipe frequency during 5 min period was measured and compared between CAP- and VEH-injected animals. ***p < 0.001, t = 10.74 (unpaired t-test, n = 4)

AFVN make vagovagal connections and innervate peripheral target organs through efferent fibers. To examine whether hVNS-induced activation of afferent fibers affects on anti-inflammatory responses in the liver, we set out to study the production of inflammatory cytokines after eliminating afferent vagus nerve activity. First, in order to identify NG neurons connected to the liver, we applied DiI into the hepatic vagal branch and found that a certain portion of NG neurons as identified by immunofluorescence staining of NF-200 proteins were retrogradely labeled with DiI (Fig. 6a). To investigate whether NG neurons are required for hVNS-mediated anti-inflammatory reaction, we attempted to remove NG sensory neurons by low- or high-dose of CAP administration (CAP-L or CAP-H). NG sensory neurons responding to CAP were identified by immunolabeling of TRPV-1 and additional labeling of P2X2 purinergic receptors. TRPV1-positive sensory neurons were colocalized with P2X2-labeled NG neurons in the vehicle control (marked arrows), and they were shrunken or weakly stained after CAP treatments (Fig. 6b). The number of TRPV1-positive neurons was slightly reduced by CAP-L and further decreased by CAP-H treatment (Fig. 6c). The number of cells expressing active (cleaved) form of caspase3 was significantly increased by CAP in NG neurons (Fig. 6d, e), indicating apoptotic death of NG neurons by CAP. Collectively, CAP-H was effective at eliminating CAP-responding NG neurons and thus was used for the rest of the present study. Systemic administration of CAP-H resulted in significant increases in eye-wiping behavior in CAP-H-treated groups compared to vehicle control (Fig. 6f), suggesting the behavioral effects of nociceptive TRPV-1 signaling in the cornea by CAP-treated rats (Hegarty et al. 2014).

### CAP downregulates ChAT expression and hepatic production of inflammatory cytokines

**Fig. 7:**
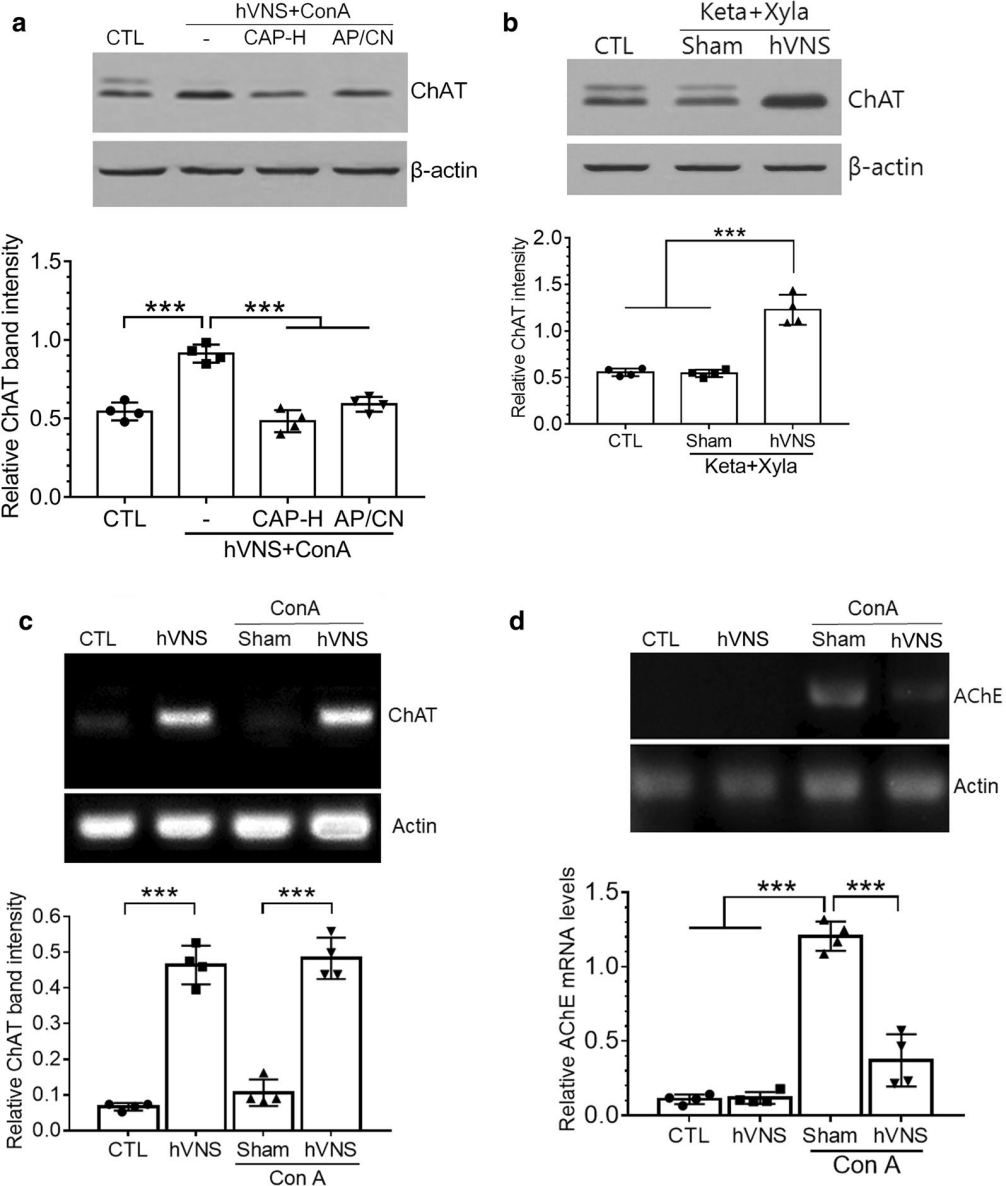
Downregulation of ChAT production by CAP. **a**, **b** Western blotting of ChAT for the lysates from DVC areas prepared from individual groups as indicated in the figure. AP/CN; a group injected with AP-5/CNQX into the DMV. Keta/Xyla; ketamine and xylazine. Western blot images in **a** and **b** are the representatives from 4 independent experiments. **c**, **d** RT-PCR for ChAT and AChE mRNAs. Amplified DNAs were analyzed by DNA gel electrophoresis and identified in all groups as a single band at 529 bp for ChAT and 307 bp for AChE. RT-PCR for actin as loading control. In **a**–**d**, one way ANOVA with Tukey post hoc is shown. ***p < 0.001 (n = 4)

To further examine that CAP-induced removal of NG neurons had an effect on efferent vagus nerve activity, we analyzed ChAT protein in the DMV after CAP treatment. The levels of ChAT protein were significantly increased in hVNS + ConA animals compared to untreated control and then reduced by CAP treatment (Fig. 7a). To explore whether CAP-mediated suppression of ChAT expression in DMV neurons can be replicated by the pharmacological blockade of signal transmission in the brain, we injected a mixture of AP5 and CNQX (labeled AP/CN in Fig. 7a) into the DMV area in hVNS + ConA animal to block glutaminergic excitatory pathway connecting afferent and efferent vagus nerve and found that ChAT level was significantly reduced to the level similar to that in CAP-H-treated animals. We also examined whether ketamine/xylazine anesthesia affected afferent vagus nerve transmission and found no difference of ChAT levels between untreated control and anesthetized groups (Fig. 7b). Having noted the variant forms of ChAT protein in terms of the molecular weights in DMV area among experimental groups (Fig. 7a, b and also Fig. 5b), we investigated the possible involvement of posttranscriptional modification of ChAT mRNA expression. A single DNA band at 529 bp (Fig. 7c upper) was identified, as expected by amplification of common-type ChAT mRNA by RT-PCR (Saito et al. 2009), implying that the variation of ChAT protein size may be derived from posttranslational, not posttranscriptional, modification. Band intensity was significantly higher in the VNS and ConA + VNS groups compared with their corresponding control groups (Fig. 7c lower). We also investigated the expression of acetylcholinesterase (AChE) mRNAs by RT-PCR in the DMV. PCR band for AChE mRNA, which was not detected from the control tissue, was clearly seen in ConA + Sham group (Fig. 7d upper) and was significantly decreased by VNS (Fig. 7d lower).

**Fig. 8:**
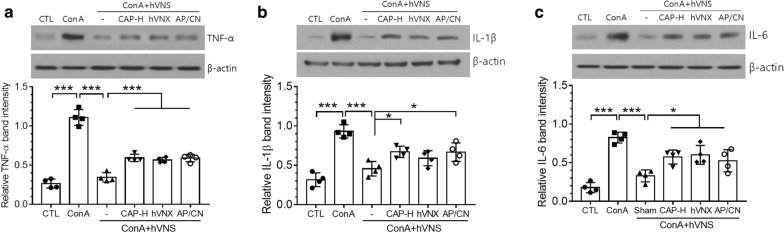
Regulation on the production of inflammatory cytokines by CAP in ConA-injected animals. Levels of inflammatory cytokines TNF-α (**a**), IL-1β (**b**), and IL-6 (**c**) in the liver tissue were analyzed by western blot analysis. All of western blot images in **a**–**c** are the representatives from 4 independent experiments, and quantification of protein bands relative to actin protein was plotted (lower panels). Western blotting for actin as internal loading control. AP/CN; a group injected with AP-5/CNQX into the DMV. In **a**–**c**, one way ANOVA with Tukey post hoc is shown. *p < 0.05, ***p < 0.001

We further examined the effects of CAP treatment on hVNS-modulated production of inflammatory cytokines in the liver. To verify whether the effect of CAP was selective on AFVN, we also analyzed hepatic inflammatory cytokines in ConA + hVNS animals given the vagotomy of hepatic branch (hVNX) or focal injection of AP-5 and CNQX inhibitors (; AP/CN in Fig. 8) into the DMV. TNF-α was strongly induced by ConA and downregulated by VNS, as demonstrated in Fig. 1. Treatment of CAP-H in ConA + hVNS animals induced an increase in TNF-α level, and the similar regulatory effects were obtained by hVNX and AP-5/CNQX injection (Fig. 8a). IL-1β, which was downregulated by hVNS following the induction by ConA, was significantly increased by CAP-H and AP-5/CNQX treatments (Fig. 8b). Finally, downrgulation of IL-6 in ConA + hVNS animals was also hampered by CAP, hVNX, and AP-5/CNQX (Fig. 8c). Thus, the manipulation of AFVN connecting to vagal efferent pathway by CAP-H, hVNX, and AP-5/CNQX appeared to exert similar suppressing activity of VNS-mediated anti-inflammation.

### hVNS increases the levels of α7 nicotinic acetylcholine receptors and phosphorylated STAT3 in the liver

**Fig. 9:**
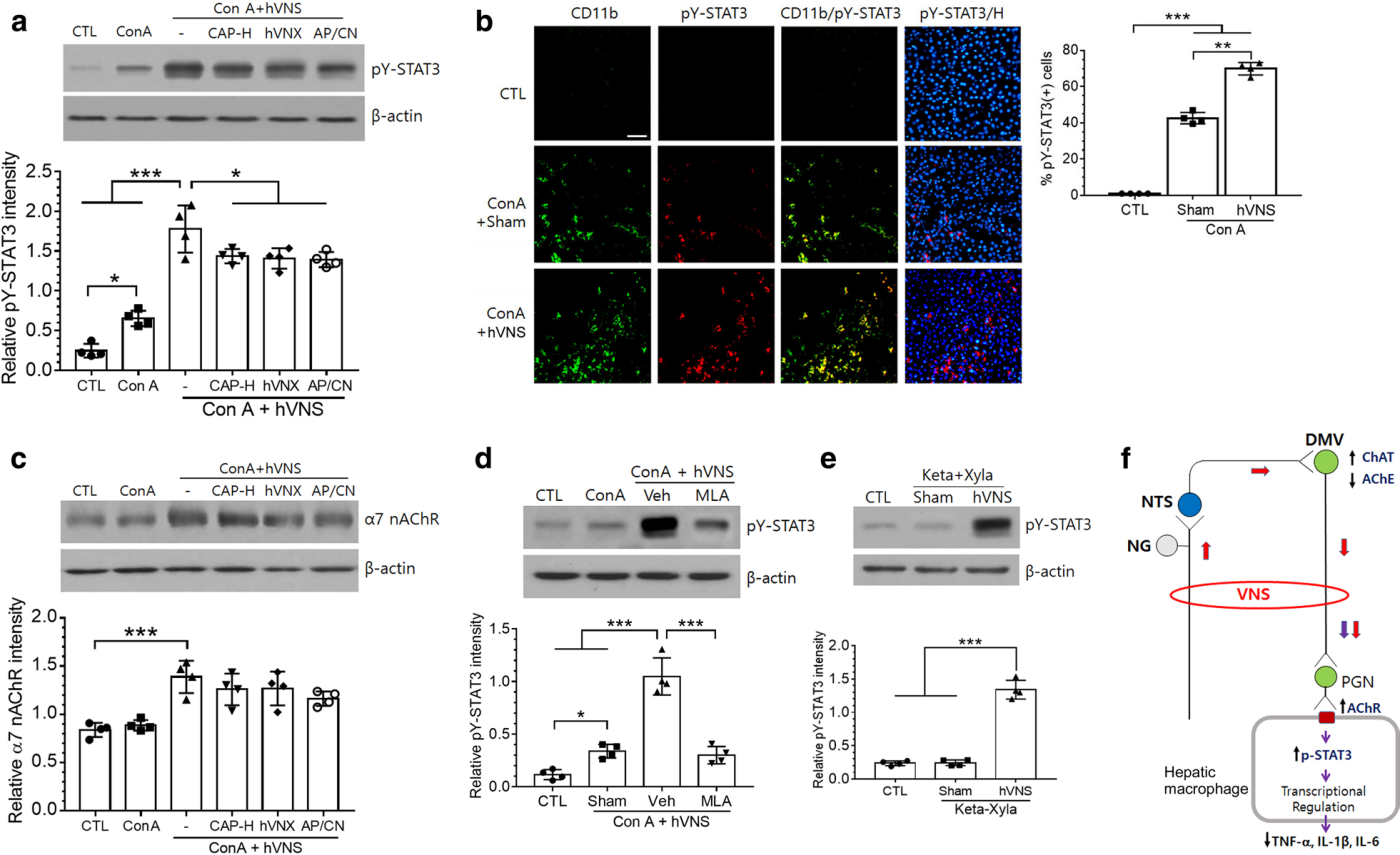
Regulation on the production of α7 nAChRs and pY-STAT3 in the liver by the inhibition of afferent vagus nerve activity in ConA-injected animals. **a** Western blot analysis of liver tissue lysates from animal groups as indicated in the figure. A representative image from 4 independent experiments is shown (upper), and a quantification of pY-STAT band intensity relative to actin is plotted (lower). Western blotting for actin as sample loading control. **b** Immunofluorescence localization of pY-STAT3 and CD11b in the liver tissue. Perinuclear localization of pY-STAT3 was identified by Hoechst nuclear staining (blue). A percentage of the number of pY-STAT3-immunostained cells relative to CD11b-labeled cells was plotted (right). **c**, **d** Western blot analysis of α7 nAChRs (**c**) and pY-STAT3 proteins (**d**, **e**) for protein lysates of liver tissue. Upper images are the representatives from 4 independent experiments. AP/CN; a group injected with AP-5/CNQX into the DMV. Keta/Xyla; ketamine/xylazine. In **a**–**e**, one way ANOVA with Tukey post hoc is shown. *p < 0.05, **p < 0.01, ***p < 0.001. Scale bar in **b** = 50 μm. **f **A schematic showing the proposed afferent vagus nerve pathway activated by VNS that contributes to cholinergic anti-inflammation in the liver. Afferent and efferent neve activity induced by VNS are shown by red and blue arrows respectively. NG; nodose ganglion, NTS; nucleus tractus solitarius, DMV; dorsal motor nucleus of the vagus nerve, PGN; postganglionic neuron

It was previously reported that JAK2-STAT3 pathway is involved in cholinergic anti-inflammation after VNS (de Jonge et al. 2005). Here in the liver tissue, pY-STAT3 was increased by ConA and the level was further increased by hVNS (Fig. 9a upper and lower). pY-STAT3 levels were significantly reduced by CAP treatment in ConA + hVNS animals, and similar effects were obtained by hVNX and AP-5/CNQX (Fig. 9a). Immunofluorescence staining showed that induced pY-STAT3 signals were mostly colocalized with CD11b-labeled cells around Hoechst-stained nuclei, showing significant increases in labeled cell numbers in both ConA + Sham and ConA + hVNS groups (Left images in Fig. 9b). Percentage of pY-STAT3-positive cells among the whole CD11b-positive cells was significantly increased in ConA + hVNS group compared to CTL and ConA + Sham groups (a plot on the right side in Fig. 9b). To explore whether α7 nAChRs are involved in VNS-induced phosphorylation of STAT3, we measured levels of α7 nAChR in the liver and found significant increase by VNS (Fig. 9c). α7 nAChR levels were slightly decreased in ConA + hVNS group of animals after treatments of CAP, hVNX, and AP-5/CNQX without showing statistical significance (p = 0.71, 0.77, 0.18 for CAP, hVNX, and AP-5/CNQX, respectively). Finally, phospho-STAT3 level was greatly suppressed in the liver tissue of ConA + hVNS group of animals which had been treated with α7 nAChR blocker MLA (Fig. 9d). Comparison of pY-STAT3 levels between untreated control and anesthetized animals with ketamine and xylazine did not showed any difference (Fig. 9e), indicating that anesthesia itself does not affect efferent nerve transmission into the liver cells.

## Discussion

In the present study, we found that hVNS regulates hepatic expression of inflammatory cytokines TNF-α, IL-1β, and IL-6 in Con-A model of hepatitis and also increases the expression of ChAT gene in the DMV neurons. Pharmacological blockades of AFVN decreased ChAT expression and suppressed anti-inflammatory effects mediated by hVNS in the liver. Thus, our data present new evidence that afferent vagus nerve activity is required for hVNS-induced anti-inflammatory response in the liver of ConA-injected animals.

T cells are activated by ConA binding and stimulate antigen-presenting cells such as Kupffer cells in the liver through the molecular interaction between T cell receptor and MHC complexes (Tiegs 1997). Inflammatory cytokines are produced from activated T cells and antigen presenting cells, causing hepatic inflammation. Given that T cells such as acetylcholine-synthesizing T cells and hepatic invariant NKT cells can be activated by adrenergic stimulation of celiac ganglionic neurons that are innervated by parasympathetic vagus nerve or from spinal sympathetic nerve activity (Wong et al. 2011; Rosas-Ballina et al. 2011), we hypothesized that the similar neuroimmune communication might be involved in the regulation of hepatic inflammation by hVNS. Here, we found that hVNS attenuated hepatic production of inflammatory cytokines TNF-α, IL-1β, and IL-6 in ConA-injected animals. Immunofluorescence images of liver tissue revealed high degree of colocalization of inflammatory cytokines with CD11b signals. Our results further showed that CD4 + and CD8 + lymphocytes recruited by ConA treatment in the liver were significantly reduced by VNS. These results indicates that macrophages and lymphocytes are recruited by ConA and produce inflammatory cytokines, and their production of inflammatory cytokines were downregulated by hVNS.

We found that c-Fos-positive DMV neurons in hVNS animals showed increased expression of ChAT, suggesting that ChAT activity may be linked to hVNS-induced cholinergic anti-inflammation. How can hVNS induce ChAT gene expression in DMV neurons? One possible pathway would be antidromic conduction of electrical stimulation of efferent fibers into neuronal cell body in the DMV. Electrical stimulation of peripheral nerve fibers after injury is known to retrogradely transport lesion signals and facilitate regenerative responses in the nucleus at gene expression level (Al-Majed et al. 2000; Mahar and Cavalli 2018). Another pathway is that the increased activity of AFVN is transmitted into NTS neurons through glutamate receptors and subsequently into DMV neurons utilizing GABA, glutamate, and norepinephrine as neurotransmitters (Andresen and Yang 1990; Travagli et al. 2006). When we administered CAP in rats in order to remove AFVN or focally injected NMDA receptor blocker AP-5 and AMPA receptor blocker CNQX, levels of ChAT that had been induced by hVNS were diminished close to baseline values of untreated control group, suggesting that the enhanced activity of AFVN by hVNS may be involved in ChAT expression in DMV neurons. However, it is unclear how neuronal excitability transmitted into the soma of DMV neurons induces ChAT gene expression. ChAT gene expression was shown to be induced in DMV neurons after vagotomy (Wang et al. 2011). Further studies are important to examine whether there are common signaling mechanisms inducing gene expression from injured axons and electrically stimulated axons.

ChAT expression generates two splice variants, common-type ChAT (cChAT; 68 kDa) and peripheral type ChAT (pChAT; 48.9 kDa) which are differentially induced in DMV neurons after vagotomy (Saito et al. 2009; Tooyama and Kimura 2000). In addition, electrophoretic mobility analysis of cChAT protein purified from rat brain showed multiple bands around 67 kDa (Dietz and Salvaterra 1980; Hersh et al. 1984). In the present study, we have identified ChAT mRNA in DMV neurons as a single amplified band by RT-PCR that corresponds to cChAT transcript. However, immunoblotting of ChAT protein in DMV lysates showed multiple bands between 67 and 72 kDa in Sham and ConA-treated groups whereas one major intense band was detected at ~ 67 kDa after hVNS. Immunoblotting for the protein lysate from cultured RAW 264.7 macrophage cells with anti-ChAT antibody did not generate any protein band, implying the absence of possible non-specific reaction of ChAT antibody with unrelated protein species. Thus, our data suggest that the process of posttranslational modification may differentially regulate so as to produce variant ChAT proteins in DMV neurons. For instance, hVNS may facilitate the reaction of proteolytic cleavage of higher molecular weight precursors of ChAT and increase the stability of processed ChAT protein, leading to the accumulation of 67 kDa of ChAT protein. Interestingly, expression of AChE mRNA in the DVC was increased by ConA and downregulated by VNS. Together with the increased production of ChAT in DMV neurons of ConA-treated animals, hVNS-induced inhibition of AChE may play a role in facilitating cholinergic neurotransmission into the peripheral target tissues such as liver and spleen and contribute to the regulation of inflammation.

Previous studies have shown that systemic or perivagal application of CAP results in degeneration of AFVN (Czaja et al. 2008; Li and Owyang 1993). CAP binds to non-selective cationic channel TRPV1, depolarize the nociceptive sensory neurons (Caterina et al. 1997), and can cause neuronal toxicity and death (Czaja et al. 2008). Here, we confirmed that the systemic administration of CAP induced apoptosis in NG neuron and deteriorated TRPV-1-positive neurons that were colocalized with P2X2-positive sensory neurons which are known to be largely associated with c-fibers of NG neurons transmitting signals via TRPV1 (Yu et al. 2005; Kwong et al. 2008). However, it was reported that CAP treatment inhibits the activation of T lymphocytes and decreases the inflammation and apoptosis of hepatocytes in ConA-treated animal (Zhang et al. 2019b). Given the difference in a time period of CAP pretreatment and injection dose (1 mg/kg for 30 min in Zhang et al. 2019b vs 200 mg/kg for 3 days in the present study) before the administration of ConA, acute CAP treatment may act on TRPV1 receptors of immune cells that are activated by ConA whereas the prolonged treatment of higher dose of CAP prior to ConA may be effective preferentially on TRPV1 receptors in sensory nerve fibers, leading to distinct pathophysiological consequences in Con-A model of hepatitis. While our data showing the similar levels of suppression of ChAT expression by the treatments of CAP and AP-5/CNQX (Fig. 7a) strongly suggest the inhibition of AFVN pathway by CAP, a possibility of direct action of CAP on vagal motor neuron affecting ChAT gene expression cannot be excluded (Browning et al. 2013). Further studies on selective blocking of vagal afferents, for instance by using surgical vagal deafferentation (Baptista et al. 2007), will be useful to further clarify this issue.

We discovered that anti-inflammatory effects of hVNS on hepatic production of inflammatory cytokines in ConA-treated animals were largely diminished by CAP treatment. Given the issue on the selectively of CAP actions to AFVN as discussed above, we investigated the production of hepatic inflammatory cytokines using two additional experimental groups in parallel: one group of animals which were injected with AP-5/CNQX into the DVC to block AFVN signals to efferent pathway, and the other group of animals in which the hVNX was applied to the hepatic branch of the vagus nerve prior to the VNS, thus delivering the efferent, but not afferent feedback loop, vagus nerve activity to the liver. Productions of hepatic TNF-α, IL-1β, and IL-6 were similar among all three groups (i.e., CAP, AP/CN, and hVNX groups) maintaining at intermediate levels higher than ConA + VNS group but lower than ConA group, supporting the notion that AFVN contributes to VNS-induced anti-inflammation in the liver.

We found that the levels of pY-STAT3 were increased by hVNS in ConA-injected animals. pY-STAT3 signals were slightly induced from CD11b-positive macrophages in the liver of ConA-injected animals, suggesting the possible involvement of pY-STAT3 activity in macrophage activation that mediates proinflammatory reactions (Wang et al. 2011). pY-STAT3 was further increased by VNS, but downregulated by CAP-H, AP-5/CNQX and hVNX. It was previously shown that the activation of JAK2-STAT2 pathway as a downstream event of α7 nAChR induces the expression of target genes such as suppressor of cytokine signaling 3 (SOCS3) and transcriptional repression of inflammatory cytokines (de Jonge et al. 2005; Yasukawa et al. 2003). Here, we demonstrate that hVNS upregulated α7 nAChR levels in the liver tissue of ConA-treated animals and the blockade of AFVN significantly decreased pY-STAT3 along with partial suppression of α7 nAChR. Importantly, a blockade of α7 nAChRs was effective at reducing the production of pY-STAT3 receptors in ConA + hVNS animals, implying the dependence of STAT3-mediated anti-inflammatory pathway on an activation of α7 nAChR. We propose possible molecular events through the feedback loop of afferent-efferent vagal pathway that may contribute to anti-inflammatory responses in the liver (Fig. 9f).

Vagal efferent fibers are known to have synaptic connection within the ganglia at the hepatic hilus and the portal space to postganglionic fibers that are extended into hepatic vasculature, bile duct, and hepatic parenchyma (McCuskey 2004; Sutherland 1964; Skaaring and Bierring 1976). It was also reported that the activation of α7 nAChRs is involved in VNS-induced anti-inflammation in hepatic cells such as Kupffer cells and hepatic resident macrophages (Wang et al. 2003; Hiramoto et al. 2008; Nishio et al. 2017; Fonseca et al. 2019). However, the sequence of events connecting between ACh-releasing efferent vagal fibers and AChR-expressing target cells in the liver remains unclear, and thus the characterization of mediators of intercellular cholinergic signaling, if any, should be of great importance to understand VNS-induced anti-inflammation in the liver.

In conclusion, we have found that acute type of hVNS has a dampening effect on hepatic inflammation and demonstrated that AFVN contributes to hVNS-induced anti-inflammation. Further technical application, for instance by using optogenetic method (Chang et al. 2015) to stimulate separately the afferent and efferent components of vagus nerve fibers, would be of great advantage to develop therapeutic strategies and also to gain insights into interaction between pathological visceral organs and the brain.

**Table 1:** Number of animals and experimental groups

Experiments	Animal groups	PCR	Western blotting	Immunofluorescence staining	FACS	Serum biochemistry
VNS experiment	Untreated CTL	5				
	ConA + Sham	4				
	ConA + hVNS	4				
	hVNS	4				
	2d ConA + Sham		5			
	2d ConA + hVNS		5			
	3d ConA + Sham		5			
	3d ConA + hVNS		5			
	4d ConA + Sham		5			
	4d ConA + hVNS		5			
	cVNS			4		
	Sham (cVNS)			4		
CAP experiment	Vehicle			4		
	CAP-L & H			4		
	CAP + Con A + hVNS		4			
AP5/CNQX injection	ConA + AP/CN + hVNS		4			
hVNX surgery	ConA + hVNX + hVNS		4			
MLA injection	ConA + Vehicle + hVNS		4			
	ConA + MLA + hVNS		4			
Ketamin injection	Ketamine + Sham		4			
	Ketamine + hVNS		4			
Retrograde tracing	DiI injection			2		

## Data Availability

The datasets used and/or analyzed during the current study are available from the corresponding author on reasonable request.

## Abbreviations

AChE: Acetylcholine esterase
AFVN: Afferent fibers of the vagus nerve
ALT: Alanine transaminase
AST: Aspartate transaminase
CAP: Capsaicin
ConA: Concanavalin A
ChAT: Choline acetyltransferase
DMV: Dorsal motor nucleus of vagus nerve
DVC: Dorsal vagal complex
hVNS: Hepatic branch of the vagus nerve
NTS: Nucleus tractus solitarius
NG: Nodose ganglion
TRPV1: Transient receptor potential vanilloid 1
CTL: Untreated control
pY-STAT3: STAT3 phosphorylation at Y705
cVNS: Cervical vagus nerve stimulation
VEH: Vehicle
CG: Celiac ganglion
PPF: Parasympathetic postganglionic fiber
SPF: Sympathetic postganglionic fiber
VNS: Vagus nerve stimulation

## References

[CR1] Al-Majed AA, Neumann CM, Brushart TM, Gordon T (2000). Brief electrical stimulation promotes the speed and accuracy of motor axonal regeneration. J Neurosci.

[CR2] Andresen MC, Yang MY (1990). Non-NMDA receptors mediate sensory afferent synaptic transmission in medial nucleus tractus solitarius. Am J Physiol.

[CR3] Baptista V, Browning KN, Travagli RA (2007). Effects of cholecystokinin-8s in the nucleus tractus solitarius of vagally deafferented rats. Am J Physiol Regul Integr Comp Physiol.

[CR4] Berthoud HR, Neuhuber WL (2000). Functional and chemical anatomy of the afferent vagal system. Auton Neurosci.

[CR5] Bockx I, Verdrengh K, Vander Elst I, van Pelt J, Nevens F, Laleman W (2012). High-frequency vagus nerve stimulation improves portal hypertension in cirrhotic rats. Gut.

[CR6] Bonaz B, Sinniger V, Pellissier S (2019). Vagus nerve stimulation at the interface of brain-gut interactions. Cold Spring Harb Perspect Med.

[CR7] Browning KN, Babic T, Holmes GM, Swartz E, Travagli RA (2013). A critical re-evaluation of the specificity of action of perivagal capsaicin. J Physiol.

[CR8] Caterina MJ, Schumacher MA, Tominaga M, Rosen TA, Levine JD, Julius D (1997). The capsaicin receptor: a heat-activated ion channel in the pain pathway. Nature.

[CR9] Chang IA, Oh MJ, Kim MH, Park SK, Kim BG, Namgung U (2012). Vimentin phosphorylation by Cdc2 in Schwann cell controls axon growth via β1-integrin activation. FASEB J.

[CR10] Chang IA, Kim KJ, Namgung U (2018). α6 and β1 Integrin heterodimer mediates Schwann cell interactions with axons and facilitates axonal regeneration after peripheral nerve injury. Neuroscience.

[CR11] Chang RB, Strochlic DE, Williams EK, Umans BD, Liberles SD (2015). Vagal sensory neuron subtypes that differentially control breathing. Cell.

[CR12] Czaja K, Burns GA, Ritter RC (2008). Capsaicin-induced neuronal death and proliferation of the primary sensory neurons located in the nodose ganglia of adult rats. Neuroscience.

[CR13] de Jonge WJ, van der Zanden EP, The FO, Bijlsma MF, van Westerloo DJ, Bennink RJ (2005). Stimulation of the vagus nerve attenuates macrophage activation by activating the Jak2-STAT3 signaling pathway. Nat Immunol.

[CR14] Dietz GW, Salvaterra PM (1980). Purification and peptide mapping of rat brain choline acetyltransferase. J Biol Chem.

[CR15] Fonseca RC, Bassi GS, Brito CC, Rosa LB, David BA, Araújo AM (2019). Vagus nerve regulates the phagocytic and secretory activity of resident macrophages in the liver. Brain Behav Immun.

[CR16] Fujii T, Mashimo M, Moriwaki Y, Misawa H, Ono S, Horiguchi K (2017). Physiological functions of the cholinergic system in immune cells. J Pharmacol Sci.

[CR17] Grundy D (2015). Principles and standards for reporting animal experiments in The Journal of Physiology and Experimental Physiology. J Physiol.

[CR18] Gu X, Huang D, Ci L, Shi J, Zhang M, Yang H (2017). Fate tracing of hepatocytes in mouse liver. Sci Rep.

[CR19] Hegarty DM, Hermes SM, Largent-Milnes TM, Aicher SA (2014). Capsaicin-responsive corneal afferents do not contain TRPV1 at their central terminals in trigeminal nucleus caudalis in rats. J Chem Neuroanat.

[CR20] Hersh LB, Wainer BH, Andrews LP (1984). Multiple isoelectric and molecular weight variants of choline acetyltransferase Artifact or real?. J Biol Chem.

[CR21] Hiramoto T, Chida Y, Sonoda J, Yoshihara K, Sudo N, Kubo C (2008). The hepatic vagus nerve attenuates Fas-induced apoptosis in the mouse liver via alpha7 nicotinic acetylcholine receptor. Gastroenterology.

[CR22] Inoue T, Abe C, Sung SS, Moscalu S, Jankowski J, Huang L (2016). Vagus nerve stimulation mediates protection from kidney ischemia-reperfusion injury through α7nAChR+ splenocytes. J Clin Invest.

[CR23] Izumi T, Imai J, Yamamoto J, Kawana Y, Endo A, Sugawara H (2018). Vagus-macrophage-hepatocyte link promotes post-injury liver regeneration and whole-body survival through hepatic FoxM1 activation. Nat Commun.

[CR25] Krenkel O, Tacke F (2017). Liver macrophages in tissue homeostasis and disease. Nat Rev Immunol.

[CR26] Kwong K, Kollarik M, Nassenstein C, Ru F, Undem BJ (2008). P2X2 receptors differentiate placodal vs neural crest C-fiber phenotypes innervating guinea pig lungs and esophagus. Am J Physiol Lung Cell Mol Physiol.

[CR27] Li Y, Owyang C (1993). Vagal afferent pathway mediates physiological action of cholecystokinin on pancreatic enzyme secretion. J Clin Invest.

[CR28] Li Y, Xu Z, Yu Y, Yuan H, Xu H, Zhu Q (2014). The vagus nerve attenuates fulminant hepatitis by activating the Src kinase in Kupffer cells. Scand J Immunol.

[CR29] Lim HD, Kim MH, Lee CY, Namgung U (2016). Anti-inflammatory effects of acupuncture stimulation via the vagus nerve. PLoS ONE.

[CR30] Mahar M, Cavalli V (2018). Intrinsic mechanisms of neuronal axon regeneration. Nat Rev Neurosci.

[CR31] McCuskey RS (2004). Anatomy of efferent hepatic nerves. Anat Rec A Discov Mol Cell Evol Biol.

[CR32] Metz CN, Pavlov VA (2018). Vagus nerve cholinergic circuitry to the liver and the gastrointestinal tract in the neuroimmune communicatome. Am J Physiol Gastrointest Liver Physiol.

[CR33] Nishio T, Taura K, Iwaisako K, Koyama Y, Tanabe K, Yamamoto G (2017). Hepatic vagus nerve regulates Kupffer cell activation via α7 nicotinic acetylcholine receptor in nonalcoholic steatohepatitis. J Gastroenterol.

[CR34] Ouagazzal A, Amalric M (1995). Competitive NMDA receptor antagonists do not produce locomotor hyperactivity by a dopamine-dependent mechanism. Eur J Pharmacol.

[CR35] Özdemir-Kumral ZN, Özbeyli D, Özdemir AF, Karaaslan BM, Kaytaz K, Kara MF (2017). Protective effect of nicotine on sepsis-induced oxidative multiorgan damage: role of neutrophils. Nicotine Tob Res.

[CR36] Paxinos GA, Watson C (1998). The rat brain in stereotaxic coordinates.

[CR37] Prechtl JC, Powley TL (1990). The fiber composition of the abdominal vagus of the rat. Anat Embryol (Berl).

[CR38] Reardon C, Murray K, Lomax AE (2018). Neuroimmune communication in health and disease. Physiol Rev.

[CR39] Rosas-Ballina M, Olofsson PS, Ochani M, Valdés-Ferrer SI, Levine YA, Reardon C (2011). Acetylcholine-synthesizing T cells relay neural signals in a vagus nerve circuit. Science.

[CR40] Saito A, Sato T, Okano H, Toyoda K, Bamba H, Kimura S (2009). Axotomy alters alternative splicing of choline acetyltransferase in the rat dorsal motor nucleus of the vagus nerve. J Comp Neurol.

[CR41] Saba E, Lee YS, Yang WK, Lee YY, Kim M, Woo SM (2020). Effects of a herbal formulation, KGC3P, and its individual component, nepetin, on coal fly dust-induced airway inflammation. Sci Rep.

[CR42] Shin HC, Jo BG, Lee CY, Lee KW, Namgung U (2019). Hippocampal activation of 5-HT(1B) receptors and BDNF production by vagus nerve stimulation in rats under chronic restraint stress. Eur J Neurosci.

[CR43] Skaaring P, Bierring F (1976). On the intrinsic innervation of normal rat liver. Histochemical and scanning electron microscopical studies. Cell Tissue Res.

[CR44] Sutherland SD (1964). An evaluation of cholinesterase techniques in the study of the intrinsic innervation of the liver. J Anat.

[CR45] Tiegs G (1997). Experimental hepatitis and role of cytokines. Acta Gastroenterol Belg.

[CR46] Tooyama I, Kimura HA (2000). A protein encoded by an alternative splice variant of choline acetyltransferase mRNA is localized preferentially in peripheral nerve cells and fibers. J Chem Neuroanat.

[CR47] Tracey KJ (2002). The inflammatory reflex. Nature.

[CR48] Tracey KJ (2007). Physiology and immunology of the cholinergic anti-inflammatory pathway. J Clin Invest.

[CR49] Travagli RA, Hermann GE, Browning KN, Rogers RC (2006). Brainstem circuits regulating gastric function. Annu Rev Physiol.

[CR50] Tyagi E, Agrawal R, Nath C, Shukla R (2010). Inhibitory role of cholinergic system mediated via alpha7 nicotinic acetylcholine receptor in LPS-induced neuro-inflammation. Innate Immun.

[CR51] Wang H, Yu M, Ochani M, Amella CA, Tanovic M, Susarla S (2003). Nicotinic acetylcholine receptor alpha7 subunit is an essential regulator of inflammation. Nature.

[CR52] Wang H, Lafdil F, Kong X, Gao B (2011). Signal transducer and activator of transcription 3 in liver diseases: a novel therapeutic target. Int J Biol Sci.

[CR53] Wong CH, Jenne CN, Lee WY, Léger C, Kubes P (2011). Functional innervation of hepatic iNKT cells is immunosuppressive following stroke. Science.

[CR54] Yasukawa H, Ohishi M, Mori H, Murakami M, Chinen T, Aki D (2003). IL-6 induces an anti-inflammatory response in the absence of SOCS3 in macrophages. Nat Immunol.

[CR55] Yu S, Undem BJ, Kollarik M (2005). Vagal afferent nerves with nociceptive properties in guinea-pig oesophagus. J Physiol.

[CR56] Zhang Q, Lai Y, Deng J, Wang M, Wang Z, Wang M (2019). Vagus nerve stimulation attenuates hepatic ischemia/reperfusion injury via the Nrf2/HO-1 Pathway. Oxid Med Cell Longev.

[CR57] Zhang H, Bai Y, Gao M, Zhang J, Dong G, Yan F (2019). Hepatoprotective effect of capsaicin against concanavalin A-induced hepatic injury via inhibiting oxidative stress and inflammation. Am J Transl Res.

[CR58] Zhou SY, Lu YX, Yao H, Owyang C (2008). Spatial organization of neurons in the dorsal motor nucleus of the vagus synapsing with intragastric cholinergic and nitric oxide/VIP neurons in the rat. Am J Physiol Gastrointest Liver Physiol.

